# IgG4-related sclerosing thyroiditis (Riedel-Struma): a review of clinicopathological features and management

**DOI:** 10.1007/s00428-023-03561-2

**Published:** 2023-05-19

**Authors:** Agata Czarnywojtek, Krzysztof Pietrończyk, Lester D. R. Thompson, Asterios Triantafyllou, Ewa Florek, Nadia Sawicka-Gutaj, Marek Ruchała, Maria Teresa Płazinska, Iain J. Nixon, Ashok R. Shaha, Mark Zafereo, Gregory William Randolph, Peter Angelos, Abir Al Ghuzlan, Abbas Agaimy, Alfio Ferlito

**Affiliations:** 1 Department of Pharmacology, Poznan University of Medical Sciences, 60-806 Poznan, Poland; 2 Chair and Department of Endocrinology, Metabolism and Internal Medicine, Poznan University of Medical Sciences, 60-355 Poznan, Poland; 3 Voivodal Specialistic Hospital in Olsztyn, 10-561 Olsztyn, Poland; 4 Head and Neck Pathology Consultations, Woodland Hills, CA 91364 USA; 5 Department of Pathology, Liverpool Clinical Laboratories, School of Dentistry, University of Liverpool, Liverpool, L3 5PS UK; 6 Laboratory of Environmental Research, Department of Toxicology, Poznan University of Medical Sciences, Dojazd 30 Street, 60-631 Poznan, Poland; 7 Nuclear Medicine Department, Medical University of Warsaw, 02-091 Warsaw, Poland; 8 Department of Otorhinolaryngology Head and Neck Surgery, NHS Lothian, Edinburgh, EH8 9YL UK; 9 Head and Neck Service, Memorial Sloan-Kettering Cancer Center, New York, NY 10065 USA; 10 Department of Head & Neck Surgery, MD Anderson Cancer Center, Houston, TX 77005 USA; 11 Department of Otolaryngology Head and Neck Surgery, Harvard Medical School, Boston, MA 02114 USA; 12 Section of General Surgery and Surgical Oncology, Department of Surgery, The University of Chicago, Chicago, Illinois IL 60637 USA; 13 Department of Biology and Pathology, Gustave Roussy Cancer Campus, University Paris-Saclay, 91190 Villejuif, France; 14 Institute of Pathology, University Hospital Erlangen, Friedrich-Alexander University Erlangen-Nürnberg (FAU), 91054 Erlangen, Germany; 15 Coordinator of the International Head and Neck Scientific Group, 35100 Padua, Italy

**Keywords:** Fibrosis, Hypothyroidism, Hyperthyroidism, IgG_4_- related systemic disease, Immune system, Riedel thyroiditis, Thyroidectomy, Glucocorticoid, Tamoxifen, Mycophenolate mofetil

## Abstract

We present a thorough review of the literature on Riedel thyroiditis (RT) with emphasis on aetiology, diagnosis and management, using the PubMed, Sinomed, and China National Knowledge Infrastructure databases. Although the exact aetiology of RT remains obscure, the histopathological features are consistent with a localized form of IgG_4_-related systemic disease (IgG_4_-RSD). Nevertheless, IgG4-RSD as a systemic fibroinflammatory disorder per se rarely affects the thyroid in the context of multiorgan manifestations. The initial diagnosis of RT is based on clinical history and imaging, but confirmation by histopathological examination is mandatory. In contrast to the historical surgical approach, glucocorticosteroid therapy is currently considered first line therapy, in line with the RT currently being viewed as a manifestation of, or analogous to, IgG4-RSD. For disease relapse, immunomodulatory agents (azathioprine, methotrexate, rituximab) can be used.

## Introduction

Riedel thyroiditis (RT) (Morbus Riedel, Riedel Struma, Riedel goitre) was first described in 1886 by the German surgeon Bernhard Riedel, who reported on three patients treated by thyroidectomy at the International Congress of Surgery in 1894 and 1896 [1–3]; Riedel used the descriptive term ‘*Eisenharte Struma*’ (‘iron-hard goitre’) for the condition [1]. *Iron-hard thyroiditis* and *struma lignose* have then been used interchangeably. However, similar observations had been made by Semple already in 1864 and later by Bolby in 1888, who also used similar terminology (thyroid as hard as iron). Moreover, clinicians also appreciated the rare occurrence of a hard thyroid described as a ‘wooden’ or ‘stone’ goitre [1–3].

RT tends to affect individuals aged 30 to 60 years [4–7]. There is a gender predilection with females affected three times more often than males [1, 5–8]. Thyroidectomy has traditionally been performed for this condition [9–37]. RT is a rare disease with an incidence of approximately 1:100,000 to 1.6:100,000 [38–42]. A comprehensive study conducted at the Mayo Clinic (from 1920 to 1984) identified 37 cases of RT among 57,000 thyroidectomies (0.06%) [7], but most of the literature corresponds to reports of individual cases (Table 1).

**Table 1:** Characteristics of the examined patients (sex, age, diagnosis), and treatment in Riedel thyroiditis (RT)

Lp	Study	Year	Sex (F/M)	Age (years)	Initial presentation	Thyroidectomy	Steroids	Tamoxifen	Other methods of treatment
1	Lawless et al. [43]	2022	F	36	Multinodular goitre	No	Yes	Yes	RIT
2	Er-Rahali et al. [9]	2021	F	38	Nodular goitre	Yes	Yes	No	L
3	Góralska et al. [44]	2021	F	67	Nodular goitre	No	Yes	No	No
4	Navarro-Sánchez et al. [45]	2021	F	69	Compressive neck symptoms	No	No	Yes	L
5	Pacella et al. [46]	2021	M	51	Abdominal and suprapubic pain	No	Yes	No	No
6	Shafi et al. [10]	2020	M	35	Nonspecific thyroid enlargement	Yes	Yes	Yes	L
7	Mammen et al. [11]	2019	F	51	Compressive neck symptoms	Yes	Yes	Yes	Rituximab
8	Falhammar et al. [13]	2018	F	25	Inflammation symptoms	Yes	Yes	No	MM Rituximab Azathioprine
9	Sakai et al. [14]	2018	F	66	Cough and sore throat	Yes	No	No	No
10	Simões et al. [12]	2018	F	40	Compressive neck symptoms	Yes	Yes	No	L
11	Arowolo et al. [18]	2016	M	61	Multinodular goitre	Yes	Yes	Yes	L
12	Cai et al. [16]	2016	M	45	Vasovagal reflex	Yes	No	No	L
13	Chong Xi et al. [20]	2016	F	73	Compressive neck symptoms	Yes	No	No	L
14	Darouichi et al. [15]	2016	M	45	Compressive neck symptoms	Yes	Yes	Yes	No
15	Hakeem et al. [19]	2016	F	50	Nonspecific thyroid enlargement	Yes	Yes	Yes	No
16	Rajkovaca et al. [17]	2016	F	43	Multinodular goitre	Yes	No	No	No
17	Mansberg et al. [47]	2015	F	39	Nonspecific thyroid enlargement	No	Yes	No	L
18	Bhutia et al. [21]	2014	M	60	Inflammation symptoms	Yes	No	No	No
19	Hong et al. [22]	2013	F	48	Inflammation symptoms	Yes	No	No	L, ANT
20	Lee et al. [23]	2013	F	57	Nonspecific thyroid enlargement	Yes	No	No	L
21	Pi et al. [25]	2012	F	77	Compressive neck symptoms	Yes	No	No	L
22	Wang et al. [24]	2012	F	52	Compressive neck symptoms	Yes	Yes	Yes	L
23	Eryaman et al. [26]	2011	F	46	Compressive neck symptoms	Yes	No	No	No
24	Junik et al. [27]	2011	F	44	Compressive neck symptoms	Yes	Yes	No	L
25	Zakeri et al. [47]	2011	M	51	Nonspecific thyroid enlargement	No	Yes	Yes	L
26	Pirola et al. [28]	2009	M	45	Compressive neck symptoms	Yes	No	No	L
27	Won et al. [29]	2008	F	41	Compressive neck symptoms	Yes	No	No	No
28	Cho et al. [30]	2007	F	51	Nonspecific thyroid enlargement	Yes	No	No	No
29	Dabelić et al. [31]	2003	F	46	Compressive neck symptoms	Yes	Yes	Yes	L
30	Torres-Montaner et al. [32]	2001	M	65	Compressive neck symptoms	Yes	No	No	No
31	Ozgen et al. [48]	2000	M	46	Compressive neck symptoms	No	Yes	No	No
32	Vaidya et al. [49]	1997	F	50	Inflammation symptoms	No	Yes	No	L
33	Laitt et al. [33]	1992	F	51	Nonspecific thyroid enlargement	Yes	Yes	No	L
34	Marín et al. [34]	1989	F	36	Compressive neck symptoms	Yes	No	No	L
35	Ward et al. [35]	1981	M	59	Nonspecific thyroid enlargement	Yes	No	No	No
36	Kelly et al. [36]	1979	M	26	Nonspecific thyroid enlargement	Yes	Yes	No	L
37	Turner-Warwick et al. [37]	1966	F	45	Compressive neck symptoms	Yes	No	No	No
Total	37	-	-	-	-	29	20	10	-

Legend: M – male, F – female, MM – mycophenolate mofetil, RIT – radioiodine therapy, L – levothyroxine, ANT – antibiotics

The aetiology of RT has been a topic of controversy, with genetic factors [50], viruses (e.g. Epstein-Barr) [51], and smoking [7] being raised and discussed as potential aetiological factors, but all lacking convincing evidence. More plausible is the notion that, RT likely represents an autoimmune process and a form of primary fibrogenic disease [4]. Similarities to Hashimoto’s thyroiditis and associations with other autoimmune diseases including Addison’s disease, type 1 diabetes mellitus, and pernicious anaemia have also been explored [52–55]. Currently, RT is regarded as a form of IgG_4_-related disease (IgG_4_-RSD) [56] and, in this context, may be referred to as IgG_4_-related sclerosing thyroiditis. Recently, Dahlgren et al. [57] attempted to advance the notion of a relationship between RT and IgG_4_-RSD; they examined tissues from three patients immunohistochemically and reported IgG4:IgG ratios ranging from 44–56% in two cases but only 0–20% in the remainder.

In RT, fibroblasts or fibroblast-like cells proliferate via the action of cytokines released from B- and/or T- lymphocytes [5]. Eosinophils may also have a role; degranulation of these cells has been described in RT [58], leading to ‘progressive fibrosis’ [7]. Eosinophil infiltration and extracellular MBP (major basic protein) deposition were observed by Heufelder et al. [58] in 15 of 16 patients with histologically proven Riedel’s invasive fibrous thyroiditis. Overall, the process has also been referred to as lymphoplasmacytosis with eosinophilia [59].

The fibroinflammatory process in RT involves not only the thyroid gland, but may also affect adjacent structures including parathyroids (hence frequently mimicking cT4 cancer clinically and on imaging) [59–61]; and may be accompanied by similar manifestations in organs known to be affected by the IgG4-RSD including orbital [50, 61–63], mediastinal/ thoracic (e.g., trachea, bronchi, lungs) [50, 64–68] and/or pancreatobiliary [7, 69] fibroinflammatory lesions. Bateman et al. [59] have also reported venous damage, which leads to phlebitis obliterans as seen in IgG_4_-RSD. Such systemic clinical settings are conveniently known as multifocal systemic sclerosis [50, 70].

The present article reviews the current knowledge about RT with emphasis on clinical presentation, diagnostics, and management.

## Literature review

This review was based on a literature search conducted using the PubMed, Sinomed, Embase, Medline, Cochrane, Google Scholar and China National Knowledge Infrastructure databases and covering publications from 1896 to 2022. The following terms were used in connection with RT: ‘diagnose’, ‘glucocorticoid’, ‘IgG_4_-related systemic disease’ (IgG_4_-RSD), ‘Riedel struma’, ‘retroperitoneal fibrosis’, ‘tamoxifen’ and ‘treatment’.

Out of 137 articles identified during the search for various RT, ultimately 37 cases were included. Patients’ age ranged from 26 to 77 (median, 48). Individual results of this study are presented in Table 1.

## Clinical presentation

RT manifests as a painless, hard, solid, ‘goitrous’ swelling in the mid-neck, causing tightness and trachea-oesophageal compression symptoms which may result in difficulty in breathing, dysphagia, hoarseness, aphonia, neck stiffness, coughing or a feeling of pressure [1, 5–7, 41, 42, 63, 71]. The symptoms are attributable to fibrosis which compresses and/or extends to the oesophagus, airways, recurrent laryngeal nerve, and musculature. Fibrosis of the parathyroid glands and ensuing hypoparathyroidism occur less frequently. About one-third of patients with RT suffer from ailments related to fibrosis in retroperitoneum/pancreas, mediastinum, lungs, lacrimal glands, orbit, salivary glands, and gallbladder [1, 7, 19, 39, 50–57, 59–70, 72, 73].

Occurrence of hypothyroidism varies. Percentages of about 30% have been reported [74], but the study conducted by the Mayo Clinic reported hypothyroidism in 14 (78%) of 19 patients. The remaining patients were euthyroid, but with elevated autoantibodies [antithyroglobulin (Tg-Abs) and antithyroid peroxidase (TPO-Abs) autoantibodies] [7]. Correlations between hypothyroidism and extent of fibrosis replacing the thyroid parenchyma are desirable.

Development of hyperthyroidism in the form of Graves’ disease [40, 63, 75, 76] or subacute thyroiditis [8, 30, 77, 78], in the course of RT are rare.

**Fig. 1:**
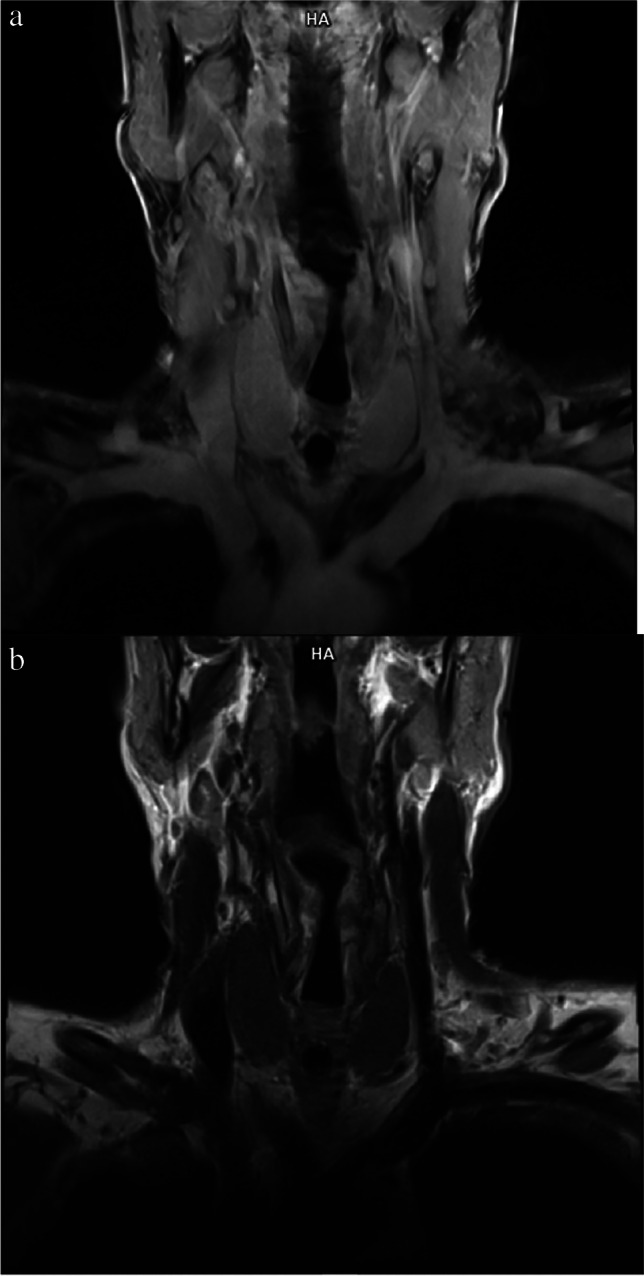
Coronal T1-weighted MRI without (**a**) and after paramagnetic contrast application (**b**). Normal thyroid with homogenous slightly hyperintense signal compared to strap muscle was visible before contrast injection and homogenous increase of the signal after injection of the contrast was noted

Clinically, it may be difficult to distinguish RT from Hashimoto’s thyroiditis or subacute thyroiditis due to comparable manifestation on imaging (Figs. 1a, b) [30, 35, 36, 40, 53, 63, 74, 76, 77, 79], and symptoms in RT may be similar to those in other thyroid diseases (Table 2) [40, 63, 75, 76].

**Table 2:** Comparative Clinical Biochemical and Imaging Features of Inflammatory Thyroid Conditions

Findings	Riedel thyroiditis	Hashimoto’s disease	Graves’ disease	De Quervain thyroiditis
Local symptoms	+	+	+	+
Systemic symptoms	±	+	+	+
Autoimmune etiology	+	+	+	±
Extrathyroidal invasion	+	-	+	-
Ophthalmopathy	-	-	+	-
Laboratory test parameters				
ESR	+	** + **	+	+
CRP	+	+	+	+
Thyroid antibodies:				
• Anti-TPO • Anti-TG • Anti-TSHR	± ± -	+ + -	+ + +	± ± -
IgG4	+	-	-	-
Hürthle cells occurrence	-	±	±	±
Additional examination				
Ultrasound appearance	Hypoechogenic	Heterogenic	Heterogenic	Hypoechogenic (In affected areas)
Doppler flow	↓	↓/N/↑	↑	↓ (In affected areas)
Radioactive Iodine uptake	↓	↓	↑	↓

Legend: ( +)-positive/excess (-)- negative/deficiency ( ±)- indefinite (↓)- decrease, (↑)- increase, (N)- no change

Malignancy may co-exist with RT [40], including papillary thyroid carcinoma, anaplastic thyroid carcinoma [80], thyroid sarcoma [32], and lymphoma [79]. Hence, care should be taken not to overlook these diseases, as their clinical and gross the presentation may significantly overlap with that of RT.

## Diagnosis

### Laboratory tests

Initial blood tests should include assessment for thyroid diseases and autoimmune processes. Complete blood count, thyroid hormone evaluation (fT4, fT3, calcitonin), thyroid-stimulating hormone (TSH), TPO-Abs, Tg-Abs and TSHR are indicated.

An increased number of white blood cells, and, rarely, erythrocytopenia [38, 40] can be seen and results may be similar to those in Hashimoto’s thyroiditis [48, 74, 76, 81–83].

The relationship between RT and IgG_4_-RSD has been addressed above. IgG_4_-RSD can involve multiple organs, though rarely the thyroid gland [84]. It is characterized by a dense lymphoplasmacytic infiltrate (with increased IgG4( +) subpopulations), obliterative phlebitis and diffuse storiform fibrosis [85]. For the first time in 2001, Hamano et al. [73] observed that sclerosing pancreatitis was associated with high serum IgG_4_ levels and response to glucocorticoid therapy. Dahlgren et al. [57] suggested that IgG_4_-RSD, in addition to RT, is also associated with other diseases such as retroperitoneal fibrosis (pancreatitis) and Küttner tumour (also see summary below). Serum IgG_4_ concentrations are usually elevated to more than 135 mg/dL in IgG_4_-RSD, but this elevation is neither necessary (found in 75% or less of affected patients) nor sufficient for diagnosis of IgG_4_-RSD [57, 84].

### Imaging

#### Ultrasonography and elastography

Ultrasonography reveals a diffuse, hypoechoic, ischaemic appearance, which is attributable to extensive fibrosis; hyperechoic bands correspond to the fibrosis [48, 74, 76, 81, 82].

Significant stiffness of the thyroid can be seen during ultrasound elastography [83].

#### ^99m^Tc thyroid scintigraphy

Isotope tests, such as thyroid scintigraphy using ^99m^Tc, show no tracer uptake within the affected tissue. 

#### Computed tomography and magnetic resonance imaging

Computed tomography (CT) shows hypodense areas within the thyroid gland, which remain unaltered after administration of a contrast agent (iodine dye) [48]. Additional imaging of the chest or abdomen may show involvement beyond the thyroid gland, indicative of a systemic process [48, 86].

Magnetic resonance imaging (MRI) reveals hypointense images by T1- and T2-weighted protocols [48].

A spectrum of slight to marked uniform enhancement can be observed following gadolinium administration [48, 82, 86–88].

Carotid artery encasement is characteristic and assists in differentiating from other thyroidopathies [7, 83].

#### Positron emission tomography (PET)

Positron emission tomography (PET) using [^18^F]fluoro-2-deoxy-D-glucose ([^18^F] FDG) clearly shows intense uptake where there are areas of inflammation–fibrosis in RT [83, 89, 90].

### Fine needle aspiration (FNA), core and open biopsies

Thyroid FNA is often inconclusive and less helpful in RT compared to other thyroid diseases. The examination may show inflamed fibrous tissue, with a keloid-like appearance, but diagnostic features such as destruction of thyroid parenchyma, storiform fibrosis, and extrathyroidal extension are only seen on core needle or open biopsy samples and in FNA specimens. An elevated number of IgG_4_ ( +) plasma cells can be observed, but overall, the features are difficult to differentiate from other disorders with similar presentation like subacute thyroiditis, the fibrous subtype of Hashimoto thyroiditis or the paucicellular subtype of anaplastic thyroid carcinoma. An open biopsy is therefore often required and can be considered optimal.

### Histopathology

Currently, histological examination remains the mainstay of the diagnosis and the decision to perform a biopsy is useful for the diagnosis of RT.

**Fig. 2:**
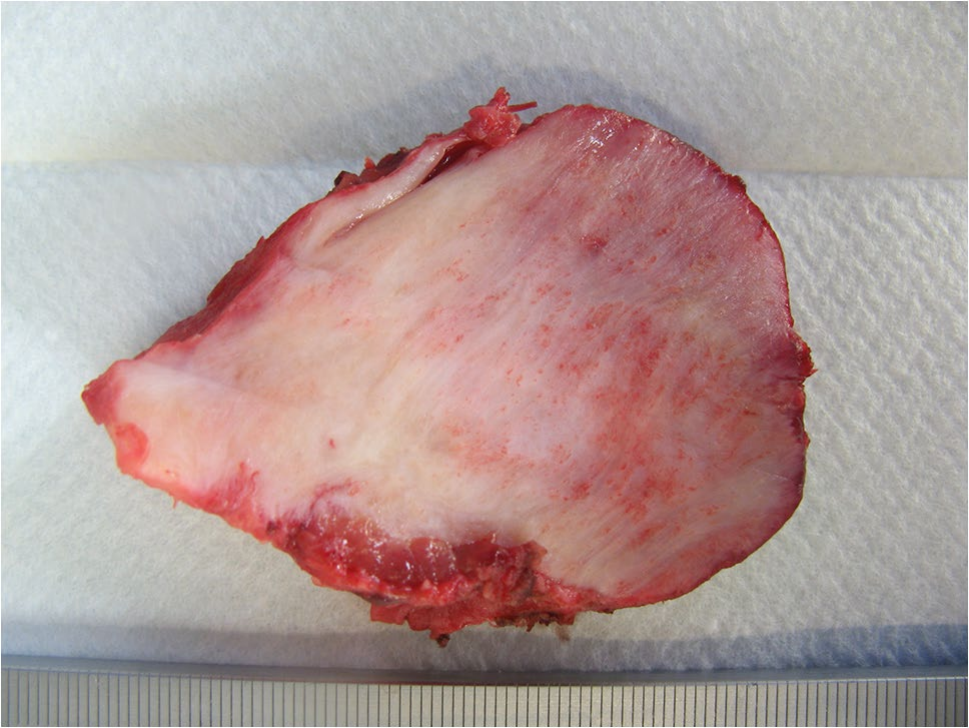
Gross section of bisected thyroidectomy specimen showing near-total replacement of the thyroid by firm fibrous tissue with entraped brownish thyroid tissue remnants at the periphgery (lower part of image)

**Fig. 3:**
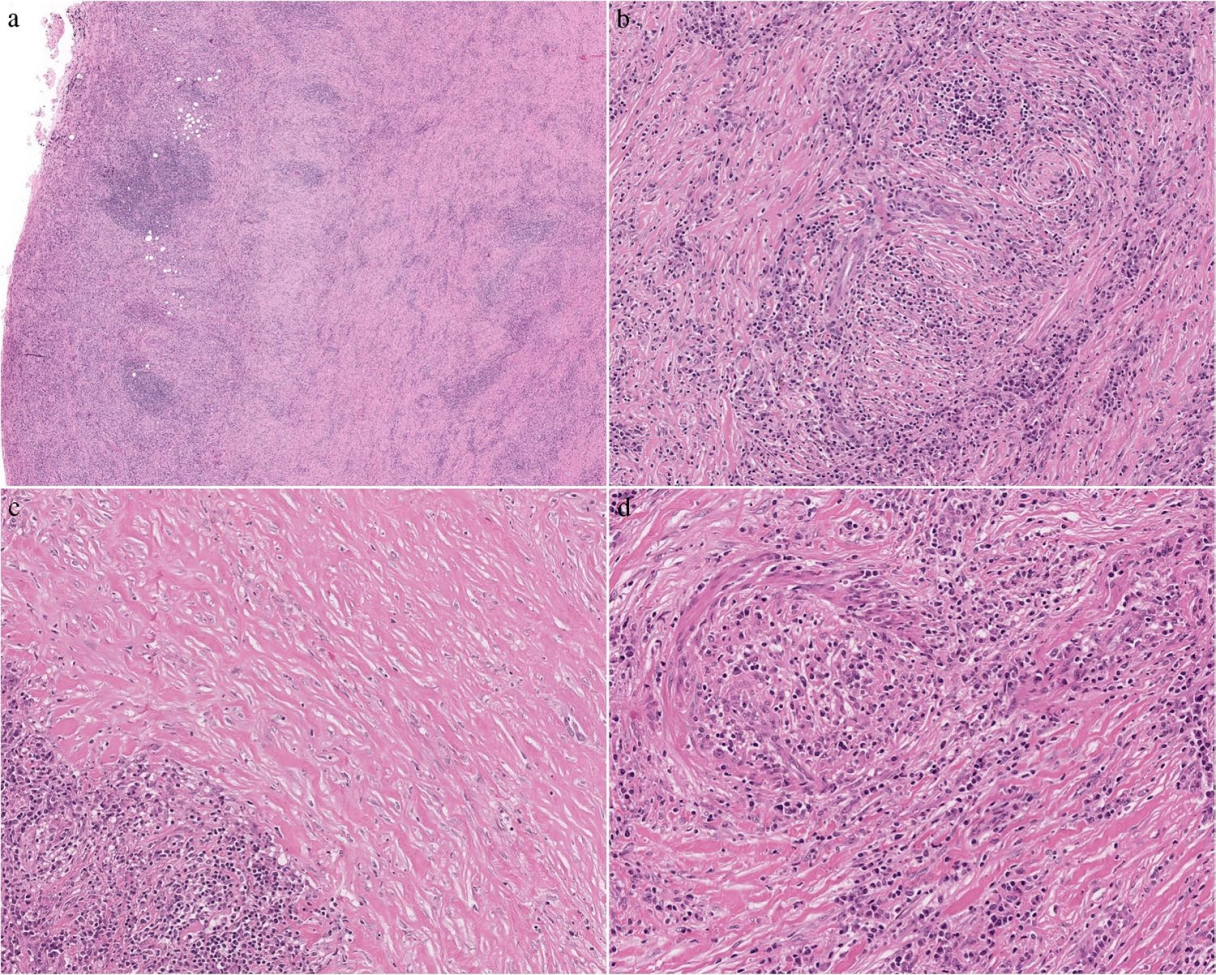
**a** The thyroid gland parenchyma has been completely overtaken by asymmetrically distributed, variably concentrated, inflammatory cell infiltrates along with fibrosis (original magnification × 20). **b** A higher power shows bundled collagen interlacing at different angles (storiform fibrosis) and predominantly lymphoplasmacytic inflammatory infiltrates. An involved small nerve is discernible in the upper right quadrant (original magnification × 40). **c** There is marked (keloid-like) fibrosis in this area of the gland where no residual thyroid parenchyma is noted. There is a lymphoplasmacytic inflammatory aggregate in the lower left corner (high power) (original magnification × 40). **d** A muscular vessel showing lumen obliteration by fibrosis with inflammation (obliterative phlebitis) is in the upper left quadrant (original magnification × 40)

Resection specimens show replacement of most of or the whole thyroid by whitish poorly marginated hard fibrous tissue with variable elastic consistency and peripheral entrapment of brownish original thyroid tissue remnants and or adjacent periglandular soft tissue (Fig. 2). Microscopic examination shows thyroid tissue with architectural distortion due to the presence of extensive fibrosis, with severe atrophy of the follicles, dense inflammatory infiltrate, and abundant plasma cells (Figs. 3a, b, c, d). Overall, RT shows the key features of IgG4-RSD including tumefactive lymphoplasmacytic inflammation, prominent storiform fibrosis and frequent obliterative angiitis (mostly phlebitis). The coexistence of elevated serum IgG4 concentrations and their presence in the histopathological examination is necessary for the diagnosis of IgG4-RD. These features are included in the revised comprehensive diagnostic (RCD) criteria for IgG4-RD. The manifestation of Riedel's disease often meets all the IgG4-RD criteria, which may indicate the co-occurrence of these diseases [91]. Immunohistochemical evaluation for IgG and IgG_4_ assists in reaching a diagnosis of RT, with more than 80 IgG_4_ ( +) plasma cells/mm^2^ and an IgG_4_/IgG ratio greater than 40% [92]. However, similar to the basic features of IgG4-RSD in other organs, these histopathological features may vary greatly based on the age of the process, from a more fibrous, paucicellular fibroinflammatory reaction to the reverse. Accordingly, in advanced stages, the predominant histopathological findings are marked tissue storiform fibrosis, absence of thyroid follicles and poor, lymphocyte-predominant cellularity. The processes characteristically extend into the perithyroidal adipose tissue, vessels (even with associated thrombosis), nerves, and even the trachea and muscles.

## Management

Standards of care are not yet established for RT, but surgery and pharmacological treatments are considered.

### Surgery

Several authors accept that surgical treatment is not indicated, at least initially [6, 7, 34]. However, particularly historically, total thyroidectomy has been attempted [9–37] to relieve compression symptoms [5, 34, 71]. Nonetheless, in the presence of significant extrathyroidal extension, surgery can be challenging and if the great vessels of the neck are encased (see above) may not be possible. After total thyroidectomy Levothyroxine is used as standard [22–25, 31, 33, 34].

If total thyroidectomy is not technically feasible, a decompressing isthmectomy maybe considered.

As the tissues in RT are very fibrous, surgical complications often occur (e.g., hypoparathyroidism or recurrence of compression symptoms) [13]. Calcium, along with calcitriol, can be usually included to counteract potential hypoparathyroidism [93].

### Pharmacological treatment

The standard approach to suppress RT is the administration of both glucocorticoids [49, 52, 77, 94–96] and tamoxifen [97].

#### Glucocorticoids

Glucocorticoids are used to treat autoimmune thyroid disease and relieve symptoms of upper respiratory tract compression, dysphonia and inflammation of the laryngeal nerve [77, 94, 98]. Standard dosages are 100 mg of prednisone daily [5]. Administration can start with lower doses (from 15 to 60 mg) and even stay at these if the response is satisfactory [9–13, 15, 18, 19, 24, 27, 31, 33, 36, 43, 44, 46–49, 78, 79, 94, 96, 99]. In the case of smokers, the dose should be increased and the therapy repeated [7]. Glucocorticosteroid therapy is often effective, but it may be followed by relapses requiring the use of immunomodulatory agents such as azathioprine, methotrexate, and, recently, rituximab [100].

#### Tamoxifen

Tamoxifen is a non-steroidal selective oestrogen-receptor modulator (SERM) of the triphenylethylene family, which include clomifene, nafoxidine, ospemifene and toremifene [101, 102]; and is structurally derived from diethylstilbestrol-type oestrogens and antioestrogens, such as chlorotrianisene and ethamoxytriphetol. Clomiphene was synthesized initially and then tamoxifen was developed [103–105].

Side effects include an increase in triglyceride concentration, which may slightly increase the risk of pulmonary embolism, deep vein thrombosis, or stroke [68]. Cases of hepatotoxicity have been observed with a long-term use [106]. Tamoxifen may also cause non-alcoholic fatty liver disease in overweight and obese females [69].

Currently, no acute overdose of tamoxifen has been observed [10, 11, 15, 18, 19, 24, 31, 43, 45, 47, 78, 97, 107–110]. It is noted that tamoxifen is used in other inflammatory conditions related to multifocal fibrosis [108]. It should be mentioned that this drug is primarily administered in breast cancer [32, 41, 42], dysmenorrhoea [52], gynaecomastia [56, 111], infertility [55], and early puberty-like bone maturation (in cases of females with precocious puberty) [57, 112] and McCune-Albright syndrome [57, 64, 100].

#### Mycophenolate mofetil

Mycophenolate mofetil (MM) is the 2-morpholinoethyl ester of mycophenolic acid (MPA), which suppresses the immune system by cytostatically affecting T- and B-lymphocytes. MPA selectively and reversibly inhibits inosine monophosphate dehydrogenase, which is involved in the synthesis of the guanosine nucleosides necessary for the assembly of DNA. It does not, however, affect cytokine synthesis and does not reduce the activity of neutrophils [113]. MM is used to prevent acute rejection of organ transplants (heart, liver, kidney) in combination with cyclosporine and corticosteroids in allogeneic transplant recipients [114, 115]. MM is also used in RT in combination with rituximab, as MPA alone is too weak [13].

### Other methods of treatment

Management of RT relapses after glucocorticosteroid therapy has been addressed above.

In the event of hyperthyroidism, radioiodine therapy or, in uncontrolled cases, external beam radiotherapy may be used [43], and levothyroxine is administered when hypothyroidism occurs [9, 10, 12, 16, 18, 20, 22–25, 27, 28, 31, 33, 34, 36, 47, 49, 99, 111].

There is little information on how vitamin D levels affect RT. However, because hypoparathyroidism is a side effect of RT, vitamin D is frequently mentioned in relation to the treatment. The adverse consequences of parathyroid hormone insufficiency are eliminated by using vitamin D and calcium [4, 77, 116–118].

## Conclusion

RT is a rare disease affecting the thyroid gland and adjacent tissues, clinically frequently mimicking locally advanced (cT4) malignancy. The disease leads to gradual progressive fibrosis with compression symptoms, pain, and hypothyroidism. Extrathyroidal extension in the central neck can also lead to hypoparathyroidism and vocal cord palsy. Rarely, RT may be limited to the thyroid gland. Imaging with the use of ultrasonography [48, 74, 76, 81, 82], CT [7, 48, 86], and MRI [48] or PET [79, 81, 83] assists in assessing the extent of lesions in the thyroid and the presence of additional manifestations in other organs. Diagnosis may be difficult without biopsy and histopathological difficulties in differentiating RT from anaplastic carcinoma [80] or thyroid sarcomas are experienced [32].

Upon diagnosis of RT, it is important to search for other systemic fibrosing manifestations in IgG4-RSD target organs (parathyroid glands, salivary glands, lacrimal glands, trachea, nervous system, cardiovascular system, retroperitoneum, mediastinum, lungs, etc.). Immunohistochemistry is recommended to assess the extent of IgG4 ( +) plasma cell population. Clinical trials have shown that in nearly 95% of RT cases, there are increased serum concentrations of IgG_4_ antibodies [85]. Although serum IgG_4_ levels may have a valuable role in diagnosis and post-treatment monitoring, currently, serum IgG4 level is not regarded as a specific marker in diagnosis and management of RT. Further research is desirable to verify the sensitivity and specificity of this finding [93].

It should be emphasized that RT cannot be completely cured. Glucocorticoids (prednisone, prednisolone) continue to be the initial treatment of choice. This has an anti-inflammatory effect and reduces the size of the gland, allowing the relief of compressive symptoms. Glucocorticosteroid therapy is effective but may be followed by relapses requiring the use of immunomodulatory agents, such as azathioprine, methotrexate, and recently rituximab [11, 13, 113]. In patients with symptomatic fibro-inflammatory disease in a hypothyroid phase, levothyroxine therapy should be started, and in special cases, anti-inflammatory drugs and vitamin D should be administered [4, 77, 93, 116–118].

## Data Availability

Data sharing not applicable.

## Declarations

No tissue samples were analyzed for this study which is merely based on published literature.
